# Limited Utility of Routine Surveillance MRI Following Chemoradiation for Advanced-Stage Oropharynx Carcinoma

**DOI:** 10.1155/2010/904297

**Published:** 2010-08-31

**Authors:** Gerald T. Kangelaris, Sue S. Yom, Kim Huang, Steven J. Wang

**Affiliations:** ^1^Department of Otolaryngology-Head and Neck Surgery, University of California at San Francisco, 2233 Post Street, Box 1225, CA 94115, USA; ^2^Department of Radiation Oncology, University of California at San Francisco, CA 94143-0226, USA

## Abstract

*Objectives*. To determine the utility of routine surveillance MRI in detecting locoregional recurrence following definitive chemoradiation in advanced-stage oropharynx carcinoma. *Methods*. We identified patients with Stage III-IV oropharynx carcinoma who were treated with chemoradiation between April 2000 and September 2004 and underwent longitudinal followup care at our institution. Patient charts were retrospectively reviewed for findings on MRI surveillance imaging, clinical signs and symptoms, and recurrence. *Results*. Forty patients received a total of 229 surveillance MRI scans with a minimum follow-up of three years (mean of 5.6 scans per patient). Six patients experienced false-positive surveillance studies that resulted in intervention. Four patients experienced recurrent disease, two of whom had new symptoms or exam findings that preceded radiographic identification of disease. Surveillance MRI scans identified recurrent disease in two asymptomatic patients who were salvaged, one of whom remains free of disease at follow-up. The overall sensitivity and specificity of the MRI surveillance program were 50 and 83 percent, respectively. The mean charge to each patient for the surveillance program was approximately $10,000 annually. *Conclusion*. In oropharyngeal cancer patients who have been treated with chemoradiation, an imaging surveillance program utilizing MRI produces limited opportunity for successful salvage.

## 1. Introduction

The exact role of surveillance imaging following treatment for oropharynx cancer remains undefined. Until recently, the National Comprehensive Care Network (NCCN) and the American Head and Neck Society (AHNS) limited imaging recommendations after treatment of head and neck cancers to yearly chest radiographs [1, 2]. Despite these guidelines, a survey of head and neck surgeons conducted by Paniello et al. found heterogeneity regarding the use of post treatment locoregional imaging of head and neck cancers, including advanced imaging for surveillance of disease recurrence [3].

Multiple authors have argued in favor of post-treatment imaging in head and neck cancers. Studies have demonstrated that a baseline post-treatment study carries prognostic information regarding locoregional control [4–6] and allows for comparison to subsequent studies, making it possible to detect tumor recurrences or treatment complications with more confidence [7]. Nonetheless, the evidence for periodic routine surveillance imaging is not established [8]. Some authors argue that it may lead to earlier detection of recurrence allowing for more prompt salvage therapies and improved long-term outcomes [9]. Others believe that the longer survival rates reported with surveillance imaging protocols may be due to lead-time bias [10]. 

The NCCN recently amended their recommendations to call for cross-sectional imaging of the primary site and neck within six months of treatment completion and reimaging based on clinical signs and symptoms [11]. Commonly available choices of imaging modality include computed tomography (CT), magnetic resonance imaging (MRI), diffusion-weighted MRI, positron emission tomography (PET), and PET-CT. Debate continues regarding the relative value of each modality [12]. 

At our institution, MRI has been the preferred modality for locoregional surveillance after treatment of head and neck cancers. We previously reported our institutional experience of patients who underwent intensity-modulated radiation therapy combined with concurrent platinum-based chemotherapy for Stage III or IV oropharyngeal carcinoma between April 2000 and September 2004 [13]. This dataset represents a relatively uniform population of similarly staged and treated head and neck cancer patients. The purpose of the current investigation was to determine the utility of routine surveillance MRI to detect locoregional treatment failure for advanced-stage oropharyngeal carcinoma treated with chemoradiation.

## 2. Methods

A waiver of informed consent was granted by our institution's committee on human research to conduct this retrospective review. We identified patients with Stage III-IV oropharyngeal carcinoma who underwent chemoradiation therapy between April 2000 and September 2004 and underwent longitudinal follow-up care at the University of California, San Francisco (UCSF) Comprehensive Cancer Center. Patients who received clinical and radiographic follow-up at outside institutions were excluded. Prior to treatment, all patients underwent complete history and physical examination, panendoscopy and biopsy, CT or MRI of the head and neck region, laboratory studies and chest radiography, dental evaluation, and nutritional, speech and swallowing evaluation.

Recommended post treatment surveillance included clinical history and physical examination at a frequency of every 1-2 months for the first year, 2-3 months for the second year, 3-4 months for the third and fourth years, and 6 months for the fifth year. Chest radiograph and thyroid function studies were obtained yearly. All patients underwent a post treatment baseline MRI approximately two months after completion of chemoradiation therapy. Surveillance MRIs were performed approximately every 3-4 months in the first year post treatment and approximately every 6 months in subsequent years. All patients included in this study adhered closely to this protocol. MRI sequences included axial, coronal, and sagittal pregadolinium T1; fat-saturated axial fast spin echo T2; and fat-saturated axial and coronal postgadolinium T1. Adjunctive studies, such as PET/CT scans, were performed as clinical evaluation dictated, but were not specifically analyzed in this study. References to radiographic investigations imply MRI scans for the purposes of this study.

Patient charts were retrospectively reviewed for disease staging, treatment and surveillance information, and recurrence. For patients who experienced locoregional failure, charts were reviewed for description of recurrence, imaging and physical exam findings, and patient symptomatology at time of diagnosis. Time until treatment failure was defined as the difference from chemoradiation completion until radiographic or definitive clinical evidence of recurrence, whichever came first. Radiographic failure was defined by language within the finalized MRI report specifically implicating the presence of tumor or suggesting tumor presence by interval growth or concerning radiographic features such as signal characteristics. Additionally, for patients with clinical symptoms or questionable findings on MRI scans, review at tumor board by the multidisciplinary head and neck team routinely occurred, including the presence of an experienced head and neck radiologist. These definitions were then used to calculate MRI diagnostic accuracy measurements, including sensitivity, specificity, positive predictive value, and negative predictive value. Disease persistence versus recurrence was defined as disease identified within or after a 3-month-time frame post treatment, respectively. Invasive procedures performed within 4 months of chemoradiation completion, such as neck dissection, were considered to be an extension of primary treatment rather than investigations into disease recurrence.

Expense estimates were obtained from our institution's Department of Radiology. We based our estimates from current charge data for an MRI neck with gadolinium, which was $4,942 as of April, 2010.

## 3. Results

### 3.1. Patient Characteristics

Seventy-one patients with advanced (Stage III or IV) oropharynx carcinoma were treated with concurrent chemoradiation at UCSF between April 2000 and September 2004. Forty-three patients met our inclusion criteria of clinical and radiologic MRI imaging surveillance follow-up performed at our institution. Three of these 43 patients had malignant disease identified within three months of completion of their treatment and were considered to have persistent disease. The remaining 40 patients were considered to have been rendered disease free by their initial treatment and were the focus of the remainder of our analysis.

Our study group consisted of 35 men and 5 women with a median age of 58 years (range, 41–81 years). The primary site of disease included base of tongue (27), tonsil (11), and oropharyngeal wall (2). Table 1 provides the T-stage and N-stage distributions of the patients according to the 2002 AJCC staging classification system. The disease was Stage III in 11 patients (28%) and Stage IV in 29 patients (72%). 

Four patients experienced locoregional failure after an initial complete clinical and radiographic response to treatment. Three additional patients experienced distant metastatic disease without locoregional failure, identified at a mean of 11 months post treatment (range, 4–20 months). Table 2 displays patient characteristics for these patients.

### 3.2. Locoregional Recurrent Disease

Among the four patients with locoregional recurrence following an initial complete treatment response, two experienced local failure and two experienced regional failure. Two of the four patients experienced recurrence within 12 months of completing chemoradiation. As shown in Table 3, the mean time until detection of recurrence was 20.3 months (range, 5–40 months). 

The physical exam findings and patient symptomatology of the 4 patients with locoregional recurrence are summarized in Table 4. Surveillance MRI imaging identified recurrent disease in two completely asymptomatic patients (patients 1, 4). Routine MRI scans revealed a new, enhancing mass in the tongue base (patient 1) and a cluster of enlarged level II nodes with focal necrosis (patient 4). The first patient (patient 1) was found by MRI to have cancer recurrence five months post chemoradiation and underwent salvage resection with glossectomy and modified radical neck dissection, but died of disease within three months of surgery. The second patient (patient 4) experienced isolated cervical nodal recurrence 26 months after completion of treatment and underwent a salvage neck dissection; this patient remains free of disease at two-year follow-up. Both patients were undergoing regular clinical follow-up; patients 1 and 4 had negative head & neck examinations five and 16 weeks prior to MRI detection of tumor recurrence, respectively.

Two patients (patients 2, 3) exhibited signs and/or symptoms prior to MRI evidence of disease. Symptoms included pharyngeal discomfort with foreign body sensation (patient 2) and worsening unilateral otalgia (patient 3). The first patient (patient 2) had concerning physical exam findings in the form of tongue base mucosal irregularity; a subsequent MRI scan was suspicious for locally recurrent disease. This patient underwent brachytherapy at the site of recurrence and remained free of disease at over two-year follow-up. The other patient (patient 3) informed his physician of otalgia at a regularly scheduled clinic follow-up; a new, enlarging neck mass was noted which was subsequently confirmed on fine-needle aspiration biopsy. This patient (patient 3) was treated with additional chemotherapy, but ultimately died of progressive cervical nodal disease.

### 3.3. Surveillance Imaging

A total of 229 MRI surveillance imaging studies were performed (a mean of 5.6 studies per patient; an average of 2.0 studies per patient-year). The four patients with locoregional recurrence underwent a total of 23 studies, an average of 5.8 per patient (range, 3–9 studies). The overall sensitivity and specificity of the MRI surveillance program for advanced oropharynx carcinoma were 50 and 83 percent, respectively (Table 5). The positive predictive value was 25% and the negative predictive value was 94%. To perform a simple cost analysis of our MRI surveillance program, we used the standard charge at our institution for an MRI of the neck with gadolinium, which was $4,942 in April 2010. The total expense for the MRI surveillance program approximated $1.13 million and averaged $28,293 per patient. The average annual expense of this program was $9,794 per patient.

Six patients experienced false-positive imaging results, all of whom underwent interventions to validate the suspicious imaging scans. All patients had normal physical exams and no clinical concerns at the time of positive MRI findings. Four patients underwent panendoscopy with biopsies, and two patients underwent fine-needle aspiration biopsies. For the 4 patients with concerning findings at the primary site, MRI demonstrated concerning asymmetry with enhancement, T2 prolongation, or both; all 4 MRI reports raised the possibility of tumor recurrence and suggested direct visualization, biopsy, further radiographic imaging, or a combination thereof. MRI studies for the two patients that underwent fine needle aspiration biopsies revealed enlarging or persistently large lymph nodes. MRI reports for these 2 patients both suggested the possibility of malignancy in lymph nodes. None of these six patients complained of new or worsening symptomatology at the time of radiographic study, nor did any display concerning physical exam findings. No patient suffered complications from the testing procedures, and none has subsequently shown any evidence of recurrence with a minimum of 6-month follow-up.

## 4. Discussion

Among our cohort of patients with advanced-stage oropharynx carcinoma treated with chemoradiation therapy, a surveillance imaging program with MRI was equal to patient symptomatology and physical examination in detecting cancer recurrence. A total of 229 MRI scans were performed to identify two asymptomatic patients with cancer recurrence. Despite early detection and salvage therapy in both patients, only one patient achieved a disease-free state. This outcome mirrored the identification of cancer recurrence via clinical history and physical examination, in which two patients were identified, one of whom achieved a disease-free state following salvage therapy. The overall sensitivity of our radiographic surveillance program utilizing MRI was 50 percent. The overall sensitivity of detecting recurrence based on patient symptomatology and physical examination was also 50 percent.

Oropharynx carcinoma has several unique qualities that likely contributed to our study results. The anatomic location of the oropharynx is not easily accessible for physical examination, thus increasing the potential for radiologic imaging to detect subclinical recurrence. On the other hand, current chemoradiation treatment protocols for oropharynx cancers, particularly HPV-related tumors in nonsmokers, can achieve low recurrence and second primary rates. As previously reported, among our study population of advanced oropharynx carcinoma patients who were rendered disease free by initial concurrent chemoradiation treatment, less than 10% suffered locoregional recurrence [13]. This low recurrence rate likely reduces the benefit of any surveillance program as compared to a similar program for a cancer site with a worse prognosis.

The recent literature on imaging for head and neck squamous cell carcinoma (HNSCC) reveals a preponderance of studies on PET and PET/CT rather than other modalities such as MRI, CT, and ultrasound. PET has been reported to be a highly sensitive technique for detection of HNSCC in the postchemoradiation setting [14]. A recent report of MRI for the detection of clinically suspected persistent or recurrent HNSCC showed similar high sensitivity [15]. While MRI provides the superior soft-tissue delineation and anatomic detail, the application of PET for cancer detection is unique in relying on differential metabolism of malignant versus benign tissue. There have been few studies comparing the efficacy of PET versus MRI for the detection of recurrent HNSCC, and the best imaging modality for the detection of recurrent HNSCC remains undetermined. When the patients in our study were undergoing treatment and follow-up, PET/CT had only been recently introduced to our institution. PET/CT was only used as an adjunctive tool to MRI in our cohort and did not influence the frequency or timing of detection of recurrences by MRI. We are currently comparing the utility of PET/CT versus MRI scans for oropharyngeal cancer surveillance. However, given the high locoregional control rates for oropharynx cancer that we continue to achieve with our concurrent chemoradiation protocols, we believe it is doubtful that we will see substantially different results with PET/CT compared to what we observed with MRI in the current investigation.

Our study is limited by its overall small cohort, single institution status, and retrospective nature. Regardless, our results suggest that, for advanced-stage oropharyngeal carcinoma treated with chemoradiation, a routine imaging surveillance program with MRI offers limited benefit in detecting cancer recurrence and improving long-term outcome when compared to standard clinical follow-up. This assessment is further bolstered when considering the overall expense of a routine advanced radiographic imaging program, which averaged nearly $10,000 per patient annually, and the potential patient morbidity resultant from interventions initiated by findings identified by the imaging program. Six patients in our cohort experienced a false-positive imaging study, which led to invasive procedures to evaluate the imaging findings. Although there were no complications associated with these interventions, there can be significant emotional stress associated with a “positive” radiologic imaging report.

We acknowledge that routine surveillance imaging may be of benefit in detecting recurrence in a specific subset of patients at high risk for recurrence, but within the limitations of our study, such a cohort could not be identified. Analysis of larger, multi-institutional patient populations directed at this question could help further define the benefits and limitations of a routine imaging surveillance program following chemoradiation for advanced head and neck cancer.

## 5. Conclusion

In patients with advanced-stage oropharyngeal carcinoma treated with chemoradiation, this study suggests limited benefit from routine surveillance MRI scans for detecting cancer recurrence as compared to close clinical follow-up. Further analysis is needed to identify patients at highest risk of recurrence who would most benefit from routine surveillance imaging.

## Figures and Tables

**Table 1:** Distribution of patients by the 2002 American Joint Committee on Cancer Staging Classification.

Stage	N0	N1	N2	N3	Total
T1	0	1	5	1	7
T2	0	6	12	2	20
T3	2	4	4	0	10
T4	1	1	1	0	3
Total	3	12	22	3	40

**Table 2:** Patient characteristics, primary site, and stage of locoregional and distant treatment failures.

Locoregional recurrent disease	
Number of patients	4
Age, years	65 (range, 55–81)
Sex	Male (4)
Primary site	Base of tongue (4)
TMN stage	T1N2b (1), T2N2b (2), and T3N2b (1)

Distant metastases	

Number of patients	3
Age	62 (range, 52–68)
Sex	Male (3)
Primary site	Base of tongue (3)
TMN stage	T2N1 (2), T2N2c (1)

**Table 3:** Primary site, staging, time to recurrence, and treatment outcome of locoregional failures.

Patient no.	Primary site	Stage	Site of failure	Time to failure detection, mon.	Salvage therapy	Clinical status
Locoregional recurrent disease						

1	BOT	T2N2b	Local	5	Surgery	DOD
2	BOT	T3N2b	Local	40	Brachytherapy	Alive*
3	BOT	T1N2b	Regional	11	Chemotherapy	DOD
4	BOT	T2N2b	Regional	26	Surgery	Alive^†^

DOD, died of disease

* No evidence of disease at 26 months after high-dose-rate brachytherapy and chemotherapy

^†^ No evidence of disease at 25 months after neck dissection

**Table 4:** Patient symptomatology and physical exam findings prior to radiographic evidence of failure.

Patient no.	Symptoms	Physical exam	No. of MRIs performed until detection of recurrence
Locoregional recurrent disease			

1	None	None	2
2	Throat fullness	Tongue base irregularity	9
3	Otalgia	New neck mass	3
4	None	None	6

**Table 5:** MRI surveillance program sensitivity and specificity.

Recurrent disease detected by imaging (patients)	2
Recurrent disease not detected by imaging (patients)	2
Nonrecurrent disease with false-positive study (patients)	6
Nonrecurrent disease with negative studies (patients)	30

Sensitivity (percent)	50
Specificity (percent)	83
Positive predictive value (percent)	25
Negative predictive value (percent)	94

## References

[1] National Comprehensive Cancer Network *NCCN Clinical Practice Guidelines in Oncology, Head and Neck Cancers*.

[2] American Head and Neck Society www.ahns.info/clinicalresources/guidelines.php.

[3] Paniello RC, Virgo KS, Johnson MH, Clemente MF, Johnson FE (1999). Practice patterns and clinical guidelines for posttreatment follow-up of head and neck cancers: a comparison of 2 professional societies. *Archives of Otolaryngology—Head and Neck Surgery*.

[4] Hermans R, Pameljer FA, Mancuso AA, Parsons JT, Mendenhall WM (2000). Laryngeal or hypopharyngeal squamous cell carcinoma: can follow-up CT after definitive radiation therapy be used to detect local failure earlier than clinical examination alone?. *Radiology*.

[5] Mukherji SK, Mancuso AA, Kotzur IM (1994). Radiologic appearance of the irradiated larynx. Part II. Primary site response. *Radiology*.

[6] van den Broek GB, Rasch CRN, Pameijer FA, Peter E, van den Brekel MWM, Balm AJM (2006). Response measurement after intraarterial chemoradiation in advanced head and neck carcinoma: magnetic resonance imaging and evaluation under general anesthesia?. *Cancer*.

[7] Hermans R (2004). Post-treatment imaging of head and neck cancer. *Cancer Imaging*.

[8] Hermans R (2008). Posttreatment imaging in head and neck cancer. *European Journal of Radiology*.

[9] de Visscher AVM, Manni JJ (1994). Routine long-term follow-up in patients treated with curative intent for squamous cell carcinoma of the larynx, pharynx, and oral cavity: does it make sense?. *Archives of Otolaryngology—Head and Neck Surgery*.

[10] Schwartz DL, Barker J, Chansky K (2003). Postradiotherapy surveillance practice for head and neck squamous cell carcinoma—too much for too little?. *Head and Neck*.

[11] National Comprehensive Cancer Network *NCCN Clinical Practice Guidelines in Oncology, Head and Neck Cancers*.

[12] de Bree R, Putten LVD, Brouwer J, Castelijns JA, Hoekstra OS, René Leemans C (2009). Detection of locoregional recurrent head and neck cancer after (chemo)radiotherapy using modern imaging. *Oral Oncology*.

[13] Huang K, Xia P, Chuang C (2008). Intensity-modulated chemoradiation for treatment of stage III and IV oropharyngeal carcinoma: the University of California-San Francisco experience. *Cancer*.

[14] Subramaniam RM, Truong M, Peller P, Sakai O, Mercier G (2010). Fluorodeoxyglucose-positron-emission tomography imaging of head and neck squamous cell cancer. *American Journal of Neuroradiology*.

[15] Vandecaveye V, De Keyzer F, Nuyts S (2007). Detection of head and neck squamous cell carcinoma with diffusion weighted MRI after (chemo)radiotherapy: correlation between radiologic and histopathologic findings. *International Journal of Radiation Oncology Biology Physics*.

